# The Decimal Association

**Published:** 1905-01-28

**Authors:** E. Johnson

**Affiliations:** Secretary of the Decimal Association


### THE DECIMAL ASSOCIATION.

MR. E. JOHNSON, secretary of the Decimal Association, writes: With reference to your article on the relation of the Metric System to medicine measures which appeared in your issue of January 14th, it seems to the lay mind that, if accuracy in dispensing is essential, the domestic spoon, handy as it may be, is a snare and delusion.

The fact that the spoon, with its wide variations in different households, is so commonly used seems either to demonstrate that accuracy is unnecessary, or to sufficiently account for a share of our death rate. At any rate the domestic spoon can have as little relation to our present system of weights and measures as to the metric system and the probable introduction of the latter into this country would be a fitting occasion to devise a domestic measure that should be accurate, cheap, and unbreakable. You allude to such measures having been introduced some years ago, but which for some reason you say failed to attain popularity. Does that indicate want of popularity amongst the medical profession, or the general public?

It seems to me that it is a question for medical men to decide. When a doctor recommends the use of a certain measure for the purpose of insuring greater accuracy, it is hardly conceivable that the recommendation, especi ally in case of serious illness, would he unheeded. nd

The proposal of Dr. George Crichton to institute a measun of 20 c.cm. and to call it a metric spoonful seems a very feasible one; dispensing bottles, made to contain ten of such measures and marked in accordance, would very adequately replace those in present use.

. ·. "Hardly conceivable" as it may be, it is nevertheless a matter of experience that unless they are given in the simplest terms, a doctor's instructions for dosage are almost certain to be unheeded by the more ignorant classes of patients. There is always the danger that if the measures recommended on the bottle are not easily understood, the patient will take no notice at all of the directions, but will merely take the medicine whenever he is thirsty, or otherwise feels inclined. If patients of the lower classes had the faintest notion of accuracy it would not be necessary, as it now is, to dilute their medicine so much that it has to be dispensed in beer bottles. What is true of the lowest classes holds good in a smaller degree to those quite high up in the social scale.—ED. HOSPITAL.

